# Federally qualified health center use of the Nebraska Tobacco Quitline

**DOI:** 10.18332/tpc/113354

**Published:** 2019-11-19

**Authors:** Kelly Gonzales, Ann M. Berger, Kathryn Fiandt

**Affiliations:** 1 University of Nebraska Medical Center College of Nursing, Omaha, Nebraska, United States

**Keywords:** quitline, settings, tobacco cessation interventions, federally qualified health centers, clinical staff

## Abstract

**INTRODUCTION:**

Promoting cessation services like quitlines is important to reduce tobacco-related morbidity and mortality. A critical need exists to improve clinical staff’s awareness of tobacco quitlines and reduce barriers in recommending and referring tobacco-using patients. The purpose was to obtain information on the use of the Nebraska Tobacco Quitline (NTQ) by Federally Qualified Health Center (FQHC) clinical staff at FQHC settings with tobacco-using patients. Specific aims were: 1) identify FQHC clinical staff and setting characteristics that influence current tobacco cessation assessment and interventions, and 2) identify barriers and facilitators that influence future use of NTQ by FQHC clinical staff and settings.

**METHODS:**

This study recruited participants from seven FQHC settings. All FQHC provider and non-provider clinical staff were recruited to complete a Clinical Staff Survey. The Medical Director, Associate Medical Director, or Nurse Manager at each FQHC was asked to provide setting information through the Setting Survey. Descriptive statistics were used to report percentages and qualitative data were analyzed using pattern coding.

**RESULTS:**

The main findings include insufficient awareness and insufficient perceived effectiveness of NTQ, non-providers reporting a limited role in tobacco cessation efforts beyond assessment of tobacco use, and the lack of supportive setting processes for intervention use.

**CONCLUSIONS:**

Targeted efforts are needed to increase awareness and perceived effectiveness of NTQ, for role expansion for non-providers, and to add Electronic Health Record (EHR) prompts and e-referral capability to increase cessation interventions including NTQ referrals. Future research is suggested to better understand patient-specific barriers in using NTQ.

## INTRODUCTION

In the United States (US), tobacco use is the leading cause of preventable death, accounting for over 443000 deaths annually^1,2^. Tobacco use can lead to heart disease, cancer, pulmonary disease, adverse reproductive effects, and can exacerbate chronic health conditions. In the state of Nebraska, 75000 people suffer from at least one tobacco-related disease and 15.4% of the state’s residents smoke cigarettes^3,4^. Healthy People 2020 goals include continued efforts to reduce tobacco use, increase tobacco cessation attempts, and increase tobacco cessation counseling in office-based health settings^5^.

Tobacco cessation interventions fall into two categories: behavioral-based or pharmacological-based. Quitlines are behavioral-based, although some include pharmacological-based components^6^. The US Preventive Services Task Force (USPSTF) has a Grade A recommendation that primary care clinicians and health teams ask adults about tobacco use, advise them to stop using tobacco, provide behavioral interventions, and use pharmacotherapy approved by the Food and Drug Administration (FDA)^7^. Tobacco-use assessment is a mandated quality measure and a process for assessing tobacco use that is expected in all clinical settings^8^. A systematic review found high-quality evidence that counselling can assist with tobacco cessation, and moderate-quality evidence of a small benefit when pharmacotherapy is added as an adjunct to counseling^9^. The National Comprehensive Cancer Network asserts that high-intensity behavior therapy with multiple counseling sessions is most effective, but brief counseling is also effective^10^. All of these recommendations have significant implications for all clinical staff, including non-providers^11^.

Standard quitlines are telephone-based services that provide counseling, tips on quitting, and additional resources^6^. The Center for Disease Control and Prevention (CDC) maintains that quitlines are efficacious and cost-effective for delivering a tobacco cessation intervention to a large and diverse population but are not used by enough people. Furthermore, quitlines are minimally affected by access to care or health disparities compared to other interventions6. Nebraska Tobacco Quitline (NTQ) has provided continuous services since July 2006 but has not engaged with Federally Qualified Health Centers (FQHCs) beyond the standard level of engagement they have with all Nebraska health entities. NTQ provides users with a set number of scheduled, outbound calls between the designated Quit Coach and callers and inbound calls are unlimited. English and Spanish languages are immediately available, and a language line is available that provides up to 170 languages for callers. NTQ also offers web-based services that include information, self-help tools, interactive counseling, automated email messages and chat rooms^12^. NTQ website includes additional interventions, including cessation classes available across Nebraska and ‘apps’. A referral is not required for NTQ access although providers, non-provider clinical staff, and non-clinical staff can fax or email referrals. NTQ does not, at present, accept e-referrals from the Electronic Health Record (EHR), communicate with enrolled participants via texting or routinely provide cessation medications^12^. At various times over the last two years, depending on the availability of additional funding, NTQ has offered a two-week supply of Nicotine Replacement Therapy.

NTQ has a quit rate of 22.4%^12^ and a satisfaction rate of 87%^13^, which demonstrates that while NTQ users report satisfaction, tobacco cessation is difficult to achieve. The quit rate is calculated using the North American Quitline Consortium (NAQC) standard quit-rate calculation using the 30-day point prevalence abstinence rate collected at seven months post-registration for tobacco users who reported currently using tobacco or having quit less than 30 days prior to calling the quitline at registration, and who consent to follow up^12^. Current Nebraska goals include increasing the number of quit attempts and increasing NTQ reach to the health service network and public^13^. The CDC asserts that reach continues to be an issue for quitlines, reaching only about 1% of smokers annually^6^. Low reach is attributed to inadequate promotion and lack of awareness of quitline services. In a 2016 report, nearly 2500 people in Nebraska registered for NTQ but only 53% of participants indicated they found out about the quitline from their provider^14^. In an effort to increase health provider awareness of and referrals to NTQ from health settings, informational packets were mailed to all Nebraska prescribing providers in 2017 and call volume increased. Increasing awareness and collaborating with providers to enhance quitline utilization is an approach that is supported by research^5,6,15,16^. A systems and partnership approach between primary care and quitlines has demonstrated feasibility, effectiveness, improved adoption, and found to benefit patients, providers and community programs while also meeting the Healthy People 2020 goal of increasing tobacco cessation counseling in office-based health settings.

Considering the research-practice gap involving awareness of quitlines, this study’s purpose was to obtain information by surveys on the use of NTQ by FQHC clinical staff at FQHC settings. Our research question was why are FQHC clinical staff and settings not recommending NTQ at a higher frequency? We hypothesized that there are factors that explain the underutilization of NTQ. The specific aims of this study were: 1) identify FQHC clinical staff and setting characteristics that influence current tobacco cessation assessment and interventions, and 2) identify barriers and facilitators that influence future use of NTQ by FQHC clinical staff and settings.

## METHODS

### Framework

Integrated Promoting Action on Research Implementation in Health Services (i-PARIHS) framework was used in this study to assist in understanding the processes necessary to achieve successful implementation^17^. The i-PARIHS asserts that successful implementation is dependent upon the facilitation of the following three constructs: innovation, recipients, and context. Facilitation is the process of implementation, innovation is the intervention, recipients refer to the people who are affected by and influence implementation at the individual and collective team level, and context refers to local and larger settings. Inherent in i-PARIHS is a need to consider successful implementation from both an individual clinical staff-level and the setting-level. The i-PARIHS also supports inclusion of other clinical staff in addition to providers who are affected by and influence intervention implementation^17^.

### Design, setting and participants

This survey study recruited participants from all seven Nebraska FQHCs. FQHCs are community-based settings that receive federal funds to provide primary care in underserved areas^18^. FQHC clinical staff and settings were selected for five key reasons. First, they follow the same quality of care practice guidelines and are mandated to track health outcome disparities measures for tobacco cessation^19^. Second, they are a comprehensive primary care system offering a wide range of services. Third, they are statewide and reach urban and rural populations. Fourth, they are located in areas and serve populations where tobacco use is prevalent^16,19,20^. Fifth, they share an EHR that may have common facilitators and barriers to use NTQ and the EHR may serve as the foundation for future intervention(s). The FQHCs do not have any formal contract with NTQ beyond standard access to available resources available to all Nebraska clinical entities. This contrasts with other select clinical entities that have requested to do more than the standard access to enhance visibility of NTQ in their system.

All primary care FQHC clinical staff were recruited for the Clinical Staff Survey. Primary care clinical staff was defined as anyone who participates in direct patient care with adult populations and included providers (physicians, physician assistants, nurse practitioners and nurse midwives) and non-providers (nurses, medical assistants, health coaches, and community health workers). Providers were defined as those who diagnose and treat health conditions^21^. Excluded from this study were FQHC staff who do not participate in the delivery of primary care, those who solely provide pediatric primary care, and those who do not interface with patients regarding their health. The Medical Director, Associate Medical Director, or Nurse Manager, at each FQHC, provided setting information on the Setting Survey.

This study received approval by the University of Nebraska Medical Center (UNMC) Internal Review Board (IRB # EX-058-18). The UNMC Center for Patient, Family, and Community Engagement in Chronic Care Management (CENTRIC) supported this work by providing funds for participation incentives.

### Surveys

The Clinical Staff Survey (Appendix A, Supplementary file) and the Setting Survey (Appendix B) were developed by the Principal Investigator (PI) as no suitable surveys were identified from the literature. Surveys were based on the literature of tobacco use and cessation and the i-PARIHS frameworks^6,7,12,17^. The Clinical Staff Survey included questions on knowledge, skill and attitude, processes that influence tobacco use, assessment of tobacco use, tobacco cessation interventions, factors for current tobacco cessation intervention use, and facilitators and barriers for future use of NTQ. The Setting Survey included questions on the process for assessment of tobacco use, factors for current tobacco cessation intervention use, and facilitators and barriers for future use of NTQ. Survey questions include multiple choice, mark all that apply, Likert-scale, and free-text responses. The surveys included approximately 30 questions. Surveys were placed online using the platform SurveyMonkey (San Mateo, California).

Surveys were first reviewed for content validity and clarity by ten people who were family nurse practitioners, experts in tobacco cessation, or experts in a relevant research area. Revisions were made based upon reviewers’ input. The surveys were then tested in a neighboring state’s FQHC that was ineligible for participation in this study. The Clinical Staff Survey was reviewed by two providers and six non-provider clinical staff, of which four were medical assistants. A concerted effort was made to include medical assistants to ensure that the Clinical Staff Survey was tested by people in this role. The Setting Survey was reviewed by an Associate Medical Director. Completion time was 30 minutes for each survey. The surveys were then revised based on feedback to improve clarity and flow.

### Procedures

An explanation of the study, informed consent document, and link to the online surveys were emailed to eligible participants. Evidence of consent was passively demonstrated by survey completion. Eligible staff were emailed, up to three times, to request their participation. Data collection occurred during October and November 2018.

After survey data collection, the Clinical Staff Survey participants were invited to a follow-up interview. The interview included open-ended questions designed to better understand identified barriers and facilitators to future use of NTQ. An attempt was made to recruit at least one person from each of the participating FQHCs for the interview. Interview data collection and analysis were done concurrently. Data collection stopped when one person from each participating FQHC had been interviewed.

All survey participants received $20, except for participants from one FQHC. That FQHC indicated their clinical staff were not permitted to accept anything of monetary value, and $20 was donated to the FQHC for each completed survey. Participants who completed a survey and an interview received $40.

### Data analysis

Descriptive statistics were used to report percentages and a valid denominator. Qualitative data were analyzed using pattern coding, which identifies an emergent theme and pulls together a great deal of material into meaningful units of analysis^22^.

## RESULTS

For the Clinical Staff Survey, 25% (55/220) of eligible participants completed the survey, representing five FQHCs. Participants included providers (20/55, 36%) and non-providers [35/55, 64%; medical assistants (16/55, 29%), Registered Nurses (8/55; 15%); Licensed Practical Nurses (5/55; 9%); Certified Nursing Assistants (4/55, 7%); and Health Coaches (2/55, 4%)]. Four of seven (57%) FQHCs participated in the Setting Survey. Not all participants answered all questions on the surveys, as noted by the changing denominator presented in the following sections.

### Characteristics that influence current tobacco cessation assessment and interventions

**Figure 1:**
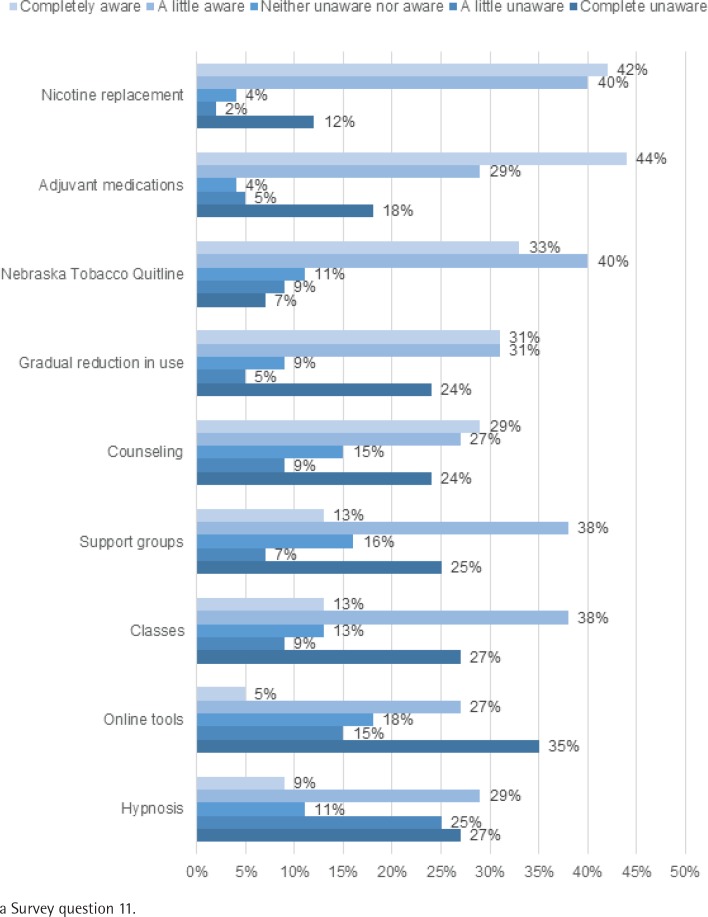
Clinical Staff Survey^a^ results of awareness of tobacco cessation interventions (n=55)

More clinical staff reported that they received formal education on assessment of tobacco use (35/55, 64%) than on tobacco cessation interventions (26/55, 47%). Figure 1 shows low awareness by clinical staff of various cessation interventions. Less than 50% reported being ‘completely aware’ of any of the interventions, including pharmacological-based nicotine replacement and adjuvant cessation medications (42–44%), NTQ (33%), and gradual reduction of tobacco use (31%). A smaller per cent of clinical staff reported being ‘a little aware’ of tobacco cessation counseling, support groups, and classes. Regarding skills reviewed during employee training, only 33% (18/55) of clinical staff indicated that assessment of tobacco use was included and only 24% (13/55) indicated tobacco cessation interventions were included.

**Figure 2:**
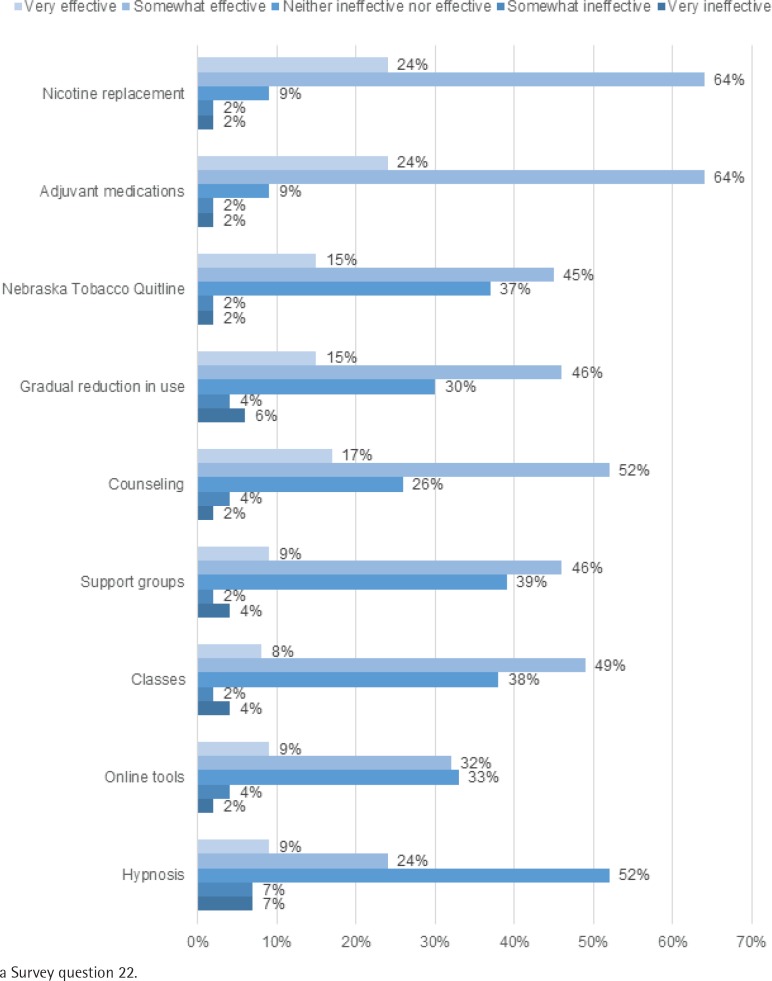
Clinical Staff Survey^a^ results of perceived effectiveness of tobacco cessation interventions (n=55)

Among clinical staff, 73% (40/55) were never tobacco users, 16% (9/55) were former tobacco users, 7% (4/55) were every day tobacco users, and 4% (2/55) were some day tobacco users (previously called occasional tobacco users). One free-text response was ‘hard to tell people to quit when I smoke’, indicating personal tobacco use influences attitude towards tobacco use and cessation. Clinical staff perceived motivation as the strongest predictor of cessation success (32/55, 58%). Figure 2 shows clinical staffs’ positive perceptions of the effectiveness of select cessation interventions. Eighty-eight per cent reported pharmacotherapy as ‘somewhat to very effective’ (88%), and fewer staff provided this rating for various behavioral interventions, including counseling (69%), gradual reduction of tobacco use (61%) and NTQ (60%).

Table 1 shows the results from the Clinical Staff Survey regarding tobacco cessation interventions currently used. Data are differentiated between Providers and Non-Providers Overall, providers reported higher use of tobacco cessation interventions compared to non-providers. Providers reported that they used the following interventions most frequently in descending order: prescribe adjuvants for cessation, set tobacco cessation goals, suggest NTQ, review reasons for tobacco cessation, and prescribe nicotine replacement therapy. Non-provider clinical staff indicated that they use the following interventions most frequently in descending order: suggest NTQ, provide education handouts/pamphlets, review general reasons for tobacco cessation, and suggest patient talk with the provider. Most providers (75%) reported suggesting NTQ, but only 40% of non-providers made this suggestion.

**Table 1:** Clinical Staff Survey^a^ results of tobacco cessation interventions currently used

*Interventions*	*Providers*			*Non-Providers*		
	*Fraction*		*%*	*Fraction*		*%*
Provide education handouts/pamphlets	7/20		35	13/35		37
Set goals related to tobacco cessation	16/20		80	8/35		23
Review general reasons for tobacco cessation	15/20		75	10/35		29
Discuss statistics related to tobacco use	4/20		20	3/35		9
Discuss specific reasons for tobacco use that relate to the individual patient	11/20		55	9/35		26
Refer to behavioral health	5/20		25	NA		
Refer to health coach	2/20		10	NA		
Refer to nurse	2/20		10	NA		
Suggest community resources	4/20		20	5/35		14
Suggest Nebraska Tobacco Quitline	15/20		75	14/35		40
Prescribe Nicotine Replacement Therapy	14/20		70	NA		
Prescribe adjuvants for cessation, i.e. varenicline or bupropion	17/20		85	NA		
Suggest talking with the provider	NA			10/35		29
Other (please specify)	1/20		5	1/35		3
	‘Assess readiness’			‘Discuss the difficulty quitting with empathy. Discuss the personal reasons why need to quit and how to make the first step’		

a a Survey questions 25–26 completed by providers (physicians, physician assistants, nurse practitioners and nurse midwives) and non-providers (nurses, medical assistants, health coaches, and community health workers).

NA: not applicable.

Table 2 shows Setting Survey results of tobacco cessation intervention currently used by the FQHCs. Data were provided by 43% (3/7) of settings. A majority of the FQHC settings indicated that provider directed interventions include the following: delivering counseling (100%), recommending or referring to cessation programs (67%), and prescribing medications to assist with cessation (67%). FQHC setting responses indicated non-provider directed interventions include the following: counseling on tobacco cessation, recommending cessation programs, and recommending NTQ. Of the three FQHC settings that answered this section on the Setting Survey, one reported that providers and two reported non-providers recommending or referring to NTQ.

**Table 2:** Setting Survey^a^ results of tobacco cessation interventions currently used

	*Response*	
	*Fraction*	*%*
**Provider directed interventions**		
Prompting patient to set tobacco cessation goals	1/3	33
Documenting tobacco cessation goals in Electronic Health Record	1/3	33
Delivering counseling on tobacco cessation	3/3	100
Recommending or referring to tobacco cessation programs, groups or counseling options	2/3	67
Recommending or referring to Nebraska Tobacco Quitline	1/3	33
Recommending tobacco cessation ‘apps’ or other mobile resources	1/3	33
Prescribing medications to assist with tobacco cessation	2/3	67
‘Order set’ available in the Electronic Health Record for tobacco cessation	0/3	0
Return or follow up visits with provider regarding tobacco cessation	1/3	33
**Non-provider clinical staff directed interventions**		
Prompting patient to set tobacco cessation goals	1/3	33
Documenting tobacco cessation goals in Electronic Health Record	1/3	33
Providing in-house counseling on tobacco cessation	2/3	67
Providing recommendations on tobacco cessation programs, groups or counseling options	2/3	67
Recommending Nebraska Tobacco Quitline	2/3	67
Recommendation tobacco cessation ‘apps’ or other mobile sources	0/3	0

a Survey questions 14–15 completed by Medical Director, Associate Medical Director, or Nurse Manager.

Table 3 presents results from the Clinical Staff Survey and Setting Survey of processes for assessment of tobacco use and current tobacco cessation intervention use. The FQHC setting leadership identified these positive processes regarding assessment of tobacco use: the presence of policies, procedures, or training; the EHR has prompts or reminders; and performance statistics are routinely reviewed to support tobacco cessation. The FQHC setting leaders indicated their EHR is NextGen (Irvine, California)^23^. The setting leaders identified the existence of a goal or benchmark related to cessation intervention documentation, that cessation interventions must be free-texted in the EHR, and the FQHC setting provides free or low-cost medications for tobacco cessation. Not all FQHC settings reported having tobacco cessation posters, pamphlets and brochures in patient common areas, nor did they have printable materials available through the EHR. Clinical Staff Survey responses regarding processes for current intervention use were less positive, with fewer than half of respondents identifying that the EHR prompts for documentation of patient-directed tobacco cessation goals or for documentation of cessation interventions. Clinical staff respondents also identified lack of time, competing demands, and lack of training as barriers for recommending tobacco cessation.

**Table 3:** Processes affecting assessment of tobacco use and current tobacco cessation intervention use

	*Clinical Staff Survey^a^*			*Setting Survey^b^*		
	*Fraction*		*%*	*Fraction*		*%*
EHR used is NextGen	-			4/4		100
Existence of health center policies, procedures of training that address assessment of tobacco use	-			4/4		100
Assessment of tobacco use is assessed when ‘rooming’ the patient	-			4/4		100
EHR provides a prompt or reminder for assessment of tobacco use	-			4/4		100
Health center reviews statistics regarding its own assessment of tobacco use at provider meetings, medical assistant meetings and other committee meetings	-			4/4		100
Existence of health center goal or benchmark related to documentation of specific tobacco cessation interventions	-			3/3		100
Health center provides free or low-cost medications to assist with tobacco cessation	-			3/3		100
Health center has tobacco cessation posters placed in common patient areas	-			2/3		67
Health center has tobacco cessation pamphlets and/or brochures placed in common patient areas	-			1/3		33
EHR has printable tobacco cessation materials available	-			1/3		33
EHR prompts for documentation of patient-directed tobacco cessation goal	26/55		47	-		
EHR prompts for documentation of a tobacco cessation treatment plan	23/55		43	-		
Tobacco cessation interventions are free-texted in the EHR	-			2/2		100
EHR does not facilitate recommending tobacco cessation	9/54		17	-		
Not enough time to accomplish recommending tobacco cessation	23/54		43	-		
Competing demands for recommending tobacco cessation	18/54		33	-		
Not enough training to accomplish recommending tobacco cessation	18/54		33	-		
Lack of awareness about recommending tobacco cessation	9/54		17	-		
Lack of resources for recommending tobacco cessation	9/54		17	-		
Tobacco cessation not the clinical priority for the health center	5/54		9	-		
Tobacco cessation not valued by health center	1/54		2	-		

a Clinical Staff Survey questions 15–16, 18.

b Setting Survey questions 4–7, 12–13, 16, 18–19.

EHR: Electronic Health Record. Hyphen denotes ‘Not asked’.

In response to Questions 23 and 28 from the Clinical Staff Survey (Appendix A), about one-half of clinical staff recommend tobacco cessation in any capacity whether it is quitting entirely or reducing current use (30/55, 55%), and less than half indicated they currently recommend NTQ (20/46, 43%). The most common staff process reasons for not currently recommending NTQ included: lack of awareness on how to recommend or refer (16/46, 35%), and lack of awareness of this resource overall (11/46, 24%).

### Barriers and facilitators that influence future use of NTQ

Table 4 displays Clinical Staff Survey and Setting Survey results of barriers and facilitators that influence future use of NTQ. Clinical staff barriers included lack of awareness, lack of time, competing demands, that the EHR doesn’t facilitate recommendation/referral, not enough training, not enough staff, and patient-specific reasons. Narrative comments for patients-pecific reasons included ‘patient not interested in quitting’ and ‘phone instability (for patients) – phone is month-to-month’. The setting barriers influencing future use included lack of awareness and patient-specific reasons. Clinical staff identified facilitators of receiving a report or summary regarding patient’s participation with NTQ, knowledge about effectiveness, additional information and/or educational in-service, streamlining educational information and referral into EHR, and provision of posters, brochures and pamphlets. The setting facilitators influencing future use included electronic referral capability within the EHR and additional information and/or education in-service.

**Table 4:** Barriers and facilitators of future use of Nebraska Tobacco Quitline

	*Clinical Staff Survey^a^*		*Setting Survey^a^*		
	*Fraction*	*%*	*Fraction*		*%*
**Barriers**				
Lack of awareness	17/51	33	2/3		67
Not enough time	16/51	31	1/3		33
Competing demands	15/41	29	1/3		33
EHR does not facilitate this	9/51	18	1/3		33
Not enough training	8/51	16	1/3		33
Not enough staff	7/51	14	1/3		33
Patient-specific reasons	4/51	8	2/3		67
Tobacco cessation is not the health center clinical priority	3/51	6	0/3		0
Lack of resources	3/51	4	0/0		0
Do not believe NTQ is effective	2/51	4	-		
Tobacco cessation not valued	0/51	0	0/3		0
Geographical/location reasons	0/51	0	0/0		0
There are no barriers	11/51	22	0/0		0
**Facilitators**				
Receiving a report or summary regarding patient’s participation with NTQ including if they complete this tobacco cessation program	39/51	76	-		
Additional information and/or educational in-service to administration or staff	26/50	52	2/3		67
Knowledge about effectiveness	27/50	54	-		
Capability for electronic referral to NTQ from within EHR	18/50	36	3/3		100
Streamline NTQ education information into EHR	20/50	40	1/3		33
Provision of posters, brochures or pamphlets on NTQ	19/50	38	1/3		33
Having one or more tobacco cessation champions	9/50	18	1/3		33
Making tobacco cessation a clinical priority for the health center	9/50	18	1/3		33

a Clinical Staff Survey questions 29–30, 33.

b Setting Survey questions 24–25.

NTQ: Nebraska Tobacco Quitline. EHR: Electronic Health Record. Hyphen denotes ‘Not asked’.

Additional qualitative data were collected through five telephone interviews with clinical staff from five different FQHCs regarding NTQ. Four themes were identified from the interviews: information, awareness, marketing, and process. All interviewees suggested the need for additional information and communication on NTQ. Other areas of information requested were additional information about the effectiveness: ‘… it would be nice to have some patient testimonials regarding its effectiveness that we could share with patients’ and ‘explain clearly what services are provided and if patients can get medication assistance’. Three of the interviewees mentioned lack of awareness as a barrier. Two interviewees mentioned the need for NTQ to engage in marketing with comments including: ‘marketing that can be for patients or staff’ and ‘people would love being given fun trinkets with info on it’. One interviewee spoke about process barriers, stating ‘love to see electronic referral’.

## DISCUSSION

The purpose of this study was to obtain information by surveys on the use of Nebraska Tobacco Quitline by FQHC clinical staff and FQHC settings, and survey findings were affirmed by qualitative telephone interviews. The main findings include insufficient awareness and insufficient perceived effectiveness of NTQ, non-providers reporting a limited role in tobacco cessation efforts beyond assessment, and lack of supportive setting processes for cessation interventions including the use of NTQ. Findings support the i-PARIHS framework including the importance of clinical staff (recipients) and setting (context) as being necessary to achieve successful implementation.

Insufficient awareness and perceived effectiveness were found to be contributing characteristics to cessation intervention use by clinical staff. Pharmacological interventions were selected most frequently by clinical staff for awareness and perceived effectiveness. Staff were not as aware of, and reported less perceived effectiveness of, NTQ and other behavioral-based interventions. Lower awareness and perceived effectiveness with behavioral-based cessation interventions are concerning because guidelines and research prioritize behavioral-based interventions over pharmacological interventions^7,9^. Authors believe the higher awareness and perceived effectiveness of pharmacological interventions reflect US trends that include pharmacotherapy as the mainstay intervention. Counseling and lifestyle changes are less frequently used by health systems or patients for a variety of reasons. NTQ offers multiple no-cost behavioral interventions and is highly efficient for FQHC clinics and their under-served patient population. Low quitline awareness is a known issue that contributes to inadequate promotion of quitline services^6^. A systems and partnership approach between primary care and quitlines has been shown to increase awareness and enhance utilization^6,15,16,24^. In 2019, NTQ began to provide patient progress reports to the referring providers but this was not available when data collection occurred.

Clinical staff and setting characteristics were identified that negatively influence tobacco cessation intervention use overall, and NTQ specifically. Providers reported using tobacco cessation interventions more frequently than non-providers (nurses, medical assistants, health coaches, and community health workers). A discrepancy was identified in which FQHC leadership reported non-providers recommend tobacco cessation interventions more than was self-reported by non-providers. Authors suspect the limited role of non-providers is due, to some extent, to confusion on who is responsible for advising cessation and providing behavioral interventions. USPSTF recommendations support the integral role of non-providers in advising tobacco cessation^7^. Clinicians and non-clinician healthcare team members can refer patients to NTQ^12^. An efficient and effective process at both the clinical staff and setting-level is to include non-providers in tobacco cessation efforts, as supported by research suggesting new roles for non-provider clinical staff including relational roles such as health coach^11^. Specific skills for the non-provider clinical staff include assisting with cessation goal setting and recommending tobacco cessation interventions such as NTQ.

There is a lack of supportive setting processes for current intervention use that impedes future use of NTQ specifically. Processes related to the EHR included the lack of prompts to document a patient-directed tobacco cessation goal and tobacco cessation interventions, and lack of facilitation for recommending tobacco cessation interventions. Processes beyond the EHR included lack of time. These findings contrast with CDC recommendations that emphasize strategic efforts to institutionalize cessation interventions within the health system and setting^6^.

### Strengths and limitations

This study has several strengths. First, this study opted for a clinical staff and setting-level survey approach, including FQHC providers and non-provider clinical staff to represent the broader system. Non-provider clinical staff are not routinely included in surveys and their responses led to new findings. Second, this study included qualitative methods to clarify survey findings and obtain additional details.

The study has some limitations. The small sample size and low survey response rate limits generalizability of the findings and introduces a possible response bias in that those who are responders may be different from non-responders. Data collection relied on self-report that is subject to recall bias. Surveys did not ask about frequency of behaviors as that would have more inherent recall bias than asking if behaviors occurred at all. There were missing data, with questions at the end of the survey answered less frequently. Finally, the surveys did not go into detail regarding patient-specific barriers.

### Implications

The implications from this study include specific areas for improvement and overall enhanced communication and collaboration between NTQ and FQHC clinical staff and settings. Clinical staff identified, through surveys and interviews, the need to increase awareness, information, and communication on NTQ. We recommend that NTQ collaborate with FQHC leadership to provide education and training in accordance with best practices emphasizing the importance of behavioral-based interventions with or without pharmacological-based interventions^5,6,9^. Tobacco cessation is difficult to achieve and a review of the efficacy of various cessation interventions, including NTQ, is indicated. A study of the target population of tobacco users may identify additional barriers to using NTQ.

There is a missed opportunity when only providers are targeted to increase the attempted quit rate and increase NTQ reach. USPSTF recommendations have implications for all clinical staff, including non-providers^11^. This implication is valuable in rural areas or those with a provider shortage.

Based on the lack of supportive setting processes that were identified and CDC recommendations^6^, we recommend adding documentation prompts within the EHR for cessation interest and cessation interventions previously or currently used, as well as adding an e-referral capability to NTQ. Enhancing e-referral capacity among state quitlines is an ongoing focus for NAQC as a strategy to increase cessation and encourage health system change^25^. Quitline e-referrals are available in over 25 other states, including states that use the same EHR as the Nebraska FQHCs^26^. These suggested process changes are supported by the i-PARIHS framework that emphasizes addressing contextual issues and improving awareness to achieve successful intervention implementation.

## CONCLUSIONS

This study provides information on the use of Nebraska Tobacco Quitline with FQHC clinical staff and settings. The main findings include the insufficient awareness and insufficient perceived effectiveness of NTQ, limited role of non-providers beyond assessment, and lack of supportive setting processes for cessation intervention use including the use of NTQ. This study supports targeted efforts to increase awareness and perceived effectiveness of NTQ, role expansion for non-providers, and adding EHR prompts and e-referral capability to increase cessation intervention use including NTQ referrals.
