# Supplementary material for: Federally qualified health center use of the Nebraska Tobacco Quitline
Source: Tob Prev Cessat. 2019 Nov 19;5:43. doi: 10.18332/tpc/113354 (PMC7205051; doi:10.18332/tpc/113354)
Supplement: Supplementary file 1 [file TPC-5-43-s1.pdf]

Appendix A: Clinical Staff Survey (completed by providers (physicians, physician assistants, nurse practitioners and nurse midwives) and non-providers (nurses, medical assistants, health coaches, and community health workers))

**Section**	**Questions**
Section 1: Demographics	1) Name of health center, 2) What is role/position, 3) How many years in the current role with the health center, 4) How many years in the current role total, and 5) personal tobacco use
Section 2: Knowledge, skill and attitude, and processes influencing assessment of tobacco use and tobacco cessation interventions	6) Previous formal education assessment of tobacco use, 7) Health center training and orientation on assessment of tobacco use, 8) Previous formal education on tobacco cessation interventions, 9) Health center training and orientation on tobacco cessation interventions, 10) Perception regarding knowledge and preparation to address tobacco cessation with patients, 11) Awareness of various tobacco cessation interventions, 12) Does participant typically assess tobacco use when with patients, 13) Does participant review tobacco use when with patients, 14) Does participant recommend tobacco cessation interventions, 15) Are tobacco cessation interventions routinely recommended by participant, 16) Does EHR prompt documentation of a patient-directed goal related to tobacco cessation, 17) Does EHR prompt development of a treatment plan related to tobacco cessation, 18) What are the factors that affect assessment of tobacco use, 19) What are factors that affect recommending tobacco cessation, 20) What is the perceived most important predictor of tobacco cessation success, 21) Perceived activeness with promotion tobacco cessation, and 22) Perceived effectiveness of various tobacco cessation interventions.
Section 3: Individual-level tobacco cessation intervention use	Questions 23- 28: 23) Does participant routinely make tobacco cessation recommendations to patients 24) Which describes participant’s role in the health center, 25) For providers: What tobacco cessation recommendations or referrals are used, 26) For non-providers: What tobacco cessation recommendations are used? 27) What are the reasons against making tobacco cessation recommendations, 28) What are the reasons for not recommending Nebraska Tobacco Quitline.
Section 4: Facilitators and barriers for future use of Nebraska Tobacco Quitline	29) What would facilitate future use of Nebraska Tobacco Quitline, 30) What are barriers to future use of Nebraska Tobacco Quitline, 31) What information would help with understanding future use of Nebraska Tobacco Quitline, 32) Interest in using Nebraska Tobacco Quitline, and 33) Would it be helpful to receive a report or summary regarding patient’s participation with Nebraska Tobacco Quitline including if they complete the program

Appendix B: Setting Survey (completed by Medical Director, Associate Medical Director or Nurse Manager)

Section	Questions
Section 1: Demographics	1) Name of health center, 2) What is the job title of the participant, 3) Does the health center have a committee or group that has focused on tobacco cessation within the last 3 years and 4) What EHR is used by the health center
Section 2: Setting processes for assessment of tobacco use and tobacco cessation interventions	5) Does the health center have policies, procedures or training that address assessment of tobacco use, 6) When is tobacco use most typically assessed, 7) Does the EHR prompt assessment of tobacco use, 8) Does the health center have any "champions" for assessing tobacco use; 9) if yes, how are they categorized (provider or non-provider clinical staff), 10) How active is the tobacco cessation "champions" in promoting tobacco cessation to patients, 11) How active are "champions" in modeling tobacco cessation behavior to health center staff, 12) Does the health center review and discuss stats on assessment of tobacco use, specifically on ensuring that tobacco use is routinely assessed during patient encounters, and 13) if yes, when/where is this done
Section 3: Setting-level tobacco cessation intervention use	14) Which non-provider clinical staff-directed tobacco cessation interventions does the health center use, 15) Which provider-directed tobacco cessation interventions does the health center use, 16) Which resources are ready available by health center for tobacco cessation, 17) Are tobacco cessation interventions routinely documented in EHR, 18) How are tobacco cessation interventions documented in EHR, 19) Is there a tobacco cessation documentation goal or benchmark, 20) Are statistics related to documentation of specific tobacco cessation interventions shared with clinical staff, 21) Is there a formal process for recommending and/or sharing information on Nebraska Tobacco Quitline, 22) if yes, describe the process, and 23) if no, what are the reasons the health center is not using Nebraska Tobacco Quitline
Section 4: Facilitators and barriers for future use of Nebraska Tobacco Quitline	24) What would facilitate future use of Nebraska Tobacco Quitline, 25) What are the barriers for future use of Nebraska Tobacco Quitline, 26) What other information would help with understanding future use of Nebraska Tobacco Quitline, 27) Is the health center interested in using Nebraska Tobacco Quitline

© 2019 Gonzales K.
